# Supplementary material for: Identification of photoperiod-induced specific miRNAs in the adrenal glands of Sunite sheep (Ovis aries)
Source: Front Vet Sci. 2022 Jul 22;9:888207. doi: 10.3389/fvets.2022.888207 (PMC9354845; doi:10.3389/fvets.2022.888207)
Supplement: Supplementary file 1 [file Data_Sheet_1.ZIP › Supplementary file/miRDeep2 file.zip!/miRDeep2 file/Know_Structure/SP42A1/oar-mir-181a-1.pdf]

miRBase precursor : oar-mir-181a-1
Total read count : 17701
oar-miR-181a read count : 17612
remaining reads : 89

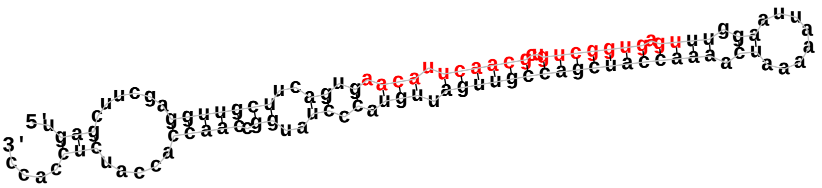

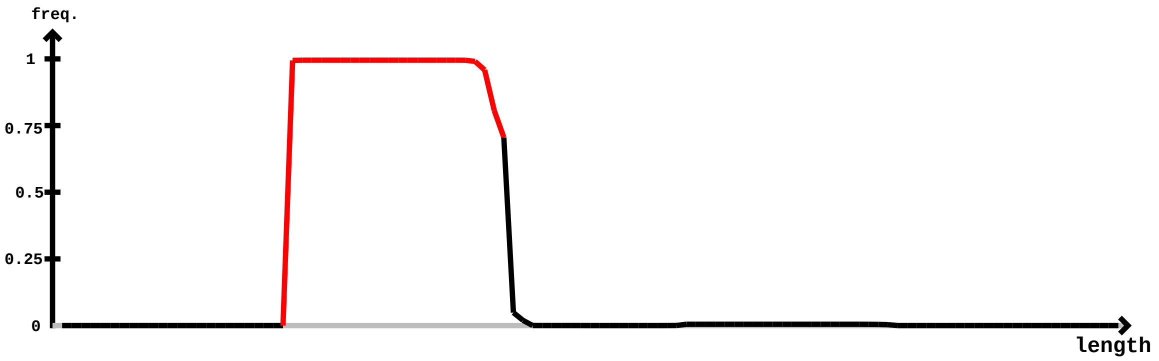


```
                       oar-miR-181a
5'- ugagcuucgagguugcuucagugaacauucaacgcugucggugaguuuggaauuaaaaaucaaaaccaucgaccguugauuguacccuauggccaaccaccaucuccacc   -3'    exp
    .(((......(((((((..((.(.(((.((((((..(((((((.((((.((........)).))))))))))))))))).))).).))..)).))))).....)))....   reads   mm   sample
    .......................aacauucaacgcugucgg.....................................................................   3       0    seq
    .......................aacauucaacgcugucggu....................................................................   77      0    seq
    .......................aacauucaacgcugucggug...................................................................   568     0    seq
    .......................aacauucaacgcugucgguga..................................................................   2694    0    seq
    .......................aacauucaacgcugucggugag.................................................................   1788    0    seq
    .......................aacauucaacgcugucggugagu................................................................   11614   0    seq
    .......................aacauucaacgcugucggugaguu...............................................................   511     0    seq
    .......................aacauucaacgcugucggugaguuu..............................................................   341     0    seq
    .......................aacauucaacgcugucggugaguuug.............................................................   2       0    seq
    .......................aacauucaacgcugucggugaguuuggaau.........................................................   1       0    seq
    .......................aacauucaacgcugucggugaguuuggaauu........................................................   1       0    seq
    ........................acauucaacgcugucggugagu................................................................   12      0    seq
    ........................acauucaacgcugucggugaguu...............................................................   1       0    seq
    .........................cauucaacgcugucggugaguuu..............................................................   1       0    seq
    ..............................................................aaaccaucgaccguugauugua..........................   1       0    seq
    ...............................................................aaccaucgaccguugauuguac.........................   3       0    seq
    ...............................................................aaccaucgaccguugauuguacc........................   1       0    seq
    ................................................................accaucgaccguugauugu...........................   4       0    seq
    ................................................................accaucgaccguugauugua..........................   4       0    seq
    ................................................................accaucgaccguugauuguac.........................   13      0    seq
    ................................................................accaucgaccguugauuguacc........................   60      0    seq
    .................................................................ccaucgaccguugauuguacc........................   1       0    seq
```
